# Remarkable Case of Hysteria Occurring in the Service of M. Guenau De Mussy

**Published:** 1849-06

**Authors:** J. H. Weir

**Affiliations:** Philadelphia


*Remarkable Case of Hysteria occurring in the service of* M. GUENAU DE MUSSY. *Hotel Dieu, Paris.* Reported by J. H. WEIR, M. D., Philadelphia.

*Courteville,* female, twenty-one years of age, of medium height, and of apparently good constitution. The catamenial discharge had been early developed, and up to the age of eighteen years she had never suffered from bad health. At that time, while engaged in washing the windows, she was seized with vertigo, (for which she could not account,) which caused her to fall from the third story of the house to the ground. This fall, according to the patient's statement, produced a fracture about the centre of the occipital bone. She remained insensible for fifteen days. Eight days after having recovered her faculties, she suddenly lost the senses of smell and sight, accompanied with a general trembling, which, having lasted for an hour and a half, left her completely paralysed, both as to motion and sensibility. The sense of taste likewise disappeared, or rather was so much perverted, that, according to the patient, every thing she put into her mouth seemed like dirt. The power of mastication was preserved, but the senses of smell, as well as the other senses, were finally lost. The patient remained in this state for three or four months, when the sight and smell returned. At this time she was seized with mental alienation, which, like the other accidents, came on suddenly, with complete loss of intelligence, the only ideas which the patient manifested, being confined to the desire of becoming a mother. This condition of the patient continued about a month, when the right leg recovered almost completely its movements and some little sensibility. The arm of the same side experienced an inverse amelioration, the sensibility being much more apparent than the movement. At the same time the right side of the face became sensible and the senses of taste and smell returned.

Two months after this, the patient while lying in bed, was seized with a violent vertigo, followed by a renewal of the complete paralysis of the right leg. After a short time, she recovered the movement of the left leg, and subsequently was enabled to move more perfectly the right one. It was not till four months after this happy change, that she began to walk about. The left arm executed some few movements but remained insensible, as did the whole of the corresponding side of the body. This improvement lasted upwards of a month, when suddenly the right arm and leg were seized, first with contraction, and finally with entire paralysis, which lasted about four months. At the same time her madness returned, lasted four months, when all these bad symptoms disappeared, and the girl was able to return home during the winter of 1839. She then enjoyed perfect health for some months, when she again lost her intellectual faculties. By the patient, this relapse was attributed to agitation resulting from an attempt made to violate her person. Her prominent idea consisted in a desire to commit suicide. She was taken to Salpetriere, which she left during the carnival of 1840, entirely cured. Entering the convent of St. Michel, she found herself so well at the end of eight months, that she again undertook the duties of a domestic. In this situation she remained for two months, when, after a slight dispute, she was attacked with severe vertigo, followed by loss of the use of the right extremities. In this state she entered the Hotel Dieu, January 1st, 1841, when the following symptoms presented themselves :—Decubitus dorsalis, respiration frequent and difficult, great anxiety of countenance. Divergent strabismus of the right eye, of the cause of which the patient is entirely ignorant. The left buccal commissure is drawn outwards, while the right has lost all power of motion. Being questioned as to the seat of pain, the patient answers with difficulty, and indicates with the left hand the præcordial region and head. Neither the ear nor the stethoscope reveal anything abnormal in the sounds of the heart, except that the pulsations are frequent and precipitate. As to the pain in the head, the patient shows the place where she asserts to have received the fracture three years ago. The pain in the præcordial region is increased upon pressure. The patient vomits everything taken into the stomach, even the Seltzer water. She appears to retain her intelligence. The whole of the right side of the body is deprived both of movement and of sensibility, and the sides of the face participate in this double paralysis, after the following manner: *on the left*, no sensibility; *on the right*, complete loss of motion. The bowels are free, there is no heat of skin, and, notwithstanding the incessant vomiting, the desire for food is not wanting. Ordered the application of two cauteries at the back of the neck—leeches over the præcordial region—diet simple.

*Jan. 2d.* The patient remains in the same state. The pain in the præcordial region is rather augmented, yet the most careful examination reveals nothing as to its cause. Some pain in the left arm, cold sweat on the face. No involuntary movements of the left side.

*January 3d.* No change in the condition of the patient. The leeching over the præcordial region was repeated. Pills of valerian were ordered, in conjunction with the warm bath, and an enema, containing four grains of assafœtida. In the evening of this day, there was a wonderful change for the better. There was a diminution of the pain in the præcordial region; a return of the sensibility of the right side, with some power of motion in the right arm. The patient answers, without much difficulty, the questions addressed to her.

This change for the better lasted two days, when, on the evening of January 5th, the resident physician, in making his visit to the ward, found the patient completely unconscious, without giving the slightest signs of life; the eyes thrown up and sensibility entirely abolished.

*Jan. 6th.* This morning the patient remains in the same state. She opens her mouth from time to time, to vomit a few mouthfuls of bile; bowels costive; ordered pills composed of valerian, castor and assafœtida; purgative enema; diet.

*Jan. 7th.* This morning the patient is gay and lively; answers all questions, and is able to move both upper and lower extremities; appetite good; pulse natural; vomiting still continues, and the patient complains very much of the cauteries. The patient continued to get better up to January 16th.

*Jan. 16th.* The patient still complains of the cauteries. The catamenia have appeared, after having been preceded by pains in the lumbar and pelvic regions.

*Jan.* 17*th.* The patient seems to be perfectly well. The catamenial flow, which were very abundant during the night, have much diminished. This evening at 8 o'clock, she was found by M. Guenau de Mussy in her bed, confined in the straight jacket. In her violent movements she succeeded twice in taking it off, and he had great difficulty in re-applying it, although assisted by the nurse, matron and one of the servants. The patient would at one time scream with the utmost violence, and then sink into a state of stupor, pronouncing now and then some incoherent words, with a convulsive trembling of the lips, like one intoxicated. The pulse was frequent and agitated, and the body was covered over with an abundant sweat. The catamenia were entirely suppressed. Ordered twenty leeches to the vulva.

*Jan.* 18*th.* Same agitation continues, with anxiety of countenance, and involuntary movements of the body. Respiration frequent; the pain in the præcordial region has returned; delirium constant, during which the patient is continually asking for her white horse to go into the country. Sensibility lost, or nearly so, over the whole body. Ordered venesection ℥xij., warm bath, and an enema of assafœtida.

*Jan.* 19*th.* Less anxiousness and agitation; pulse less frequent; continuation of the delirium; her ideas being fixed on the same subject. When questioned sharply, the patient seems to wake out of a sound sleep; looks at those around her with an astonished air; assures us she is not sick, and that she desires to return home immediately. The same treatment was continued, with the addition of an opiate at night.

Since the 19th, the patient has improved rapidly; the sensibility has returned; her ideas are under the control of reason, and she appears happy and cheerful; answers all questions, and has a good appetite.

*Feb.* 1*st.* The patient has entirely recovered; she leaves the hospital to-day.

One month afterward she entered the hospital as a domestic, but finding the duties too severe, she has gone into service. Her health continues to be good.

